# Epstein-Barr Virus-Positive Mucosal Skin Ulcer Resulting in Oral Lesions During Concomitant Use of Tacrolimus and Prednisolone

**DOI:** 10.7759/cureus.57091

**Published:** 2024-03-27

**Authors:** Itsuki Hayashi, Makoto Toida

**Affiliations:** 1 Oral and Maxillofacial Surgery, Sugita Genpaku Memorial Obama Public Hospital, Obama, JPN

**Keywords:** oral mucosa, tongue, prednisolone, tacrolimus, epstein-barr virus-positive mucocutaneous ulcers

## Abstract

Epstein-Barr virus-positive mucocutaneous ulcer (EBVMCU) is a subtype of Epstein-Barr virus-positive lymphoproliferative disease with a favorable prognosis that can develop either due to medical interventions or as a consequence of aging. Medical-onset cases caused by immunosuppressive drugs may require a reduction or discontinuation of the causative drugs. However, specific methods for drug adjustment in cases where multiple immunosuppressive drugs are used have not yet been established. Herein, we present the case of a 63-year-old man with interstitial pneumonia who developed an EBVMCU on the right side of his tongue. He was on multidrug therapy with tacrolimus and prednisolone and was treated conservatively by discontinuation of the tacrolimus and switching to prednisolone monotherapy. The lesion resolved within two months following the adjustment. This case report provides evidence that conversion to monotherapy, rather than multiple immunosuppressive drugs, is a potentially effective treatment option for EBVMCU.

## Introduction

Epstein-Barr virus-positive mucocutaneous ulcers (EBVMCUs) are a rare subtype of benign B-cell lymphoproliferative diseases. EBVMCU was first reported by Dojcinov et al. in 2010 and was designated as a novel disease group by the WHO in 2017 [1-2].

EBVMCU is slightly more common in women, and the median patient age is reported to be 66.4 years [3]. EBVMCU presents as isolated, sharply demarcated ulcers on the mucosa of the mid-pharynx (52%), skin (29%), or gastrointestinal tract (19%) [4]. Patients with EBVMCU are often in a state of medically-induced immunosuppression or age-related immune senescence [5]. Medically-induced forms of immunosuppression, including methotrexate, cyclosporine-A, azathioprine, tacrolimus, tumor necrosis factor inhibitors, mycophenolic acid, and topical steroid therapy, have all been implicated in the pathogenesis of EBVMCU [6]. Diseases that cause immunosuppression may also be implicated [5].

The etiology of EBVMCU is thought to be a proliferative disease caused by the formation of a medically- or aging-related immunosuppressive state, primarily of EBV-specific cytotoxic T-lymphocytes, with activation of dormant EBV-infected B-cells [7]. We report a rare case of EBVMCU on the tongue of a patient with interstitial pneumonia who was treated with tacrolimus and prednisolone.

## Case presentation

**Figure 1:**
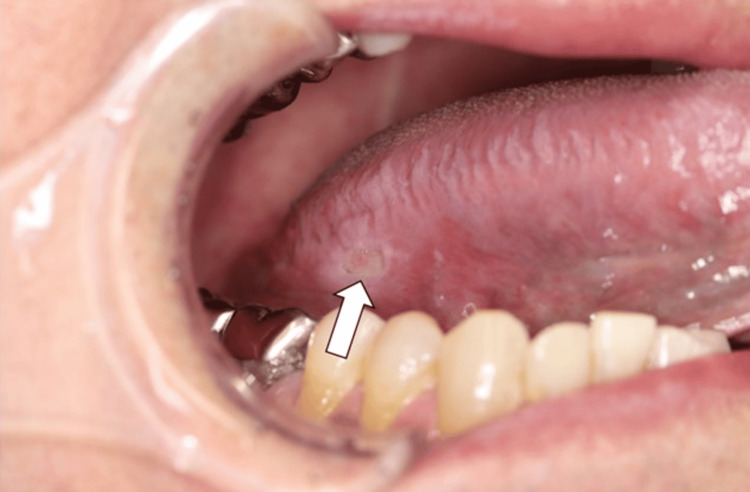
Image of an ulcer on the right lateral border of the tongue at the time of initial examination (arrow) The surface of the ulcer faces the mandibular first molars.

A 63-year-old man presented with a persistent, painful ulcer on the right lateral border of the tongue that had persisted for two weeks. He had a history of interstitial pneumonia and had been receiving combination therapy with prednisolone (20 mg once daily) and tacrolimus (3.5 mg once daily) for two years and 10 months, respectively. At the time of the patient's first visit to the department, blood tests showed a white blood cell count of 4590/µL. He denied any smoking or drinking habits. A physical examination revealed no regional or generalized lymphadenopathy. Intraoral examination revealed a well-defined, deep-seated ulcer, 1 cm in size, on the right lateral border of the tongue. There was contact pain, but no induration was palpable around the ulcer (Figure 1).

**Figure 2:**
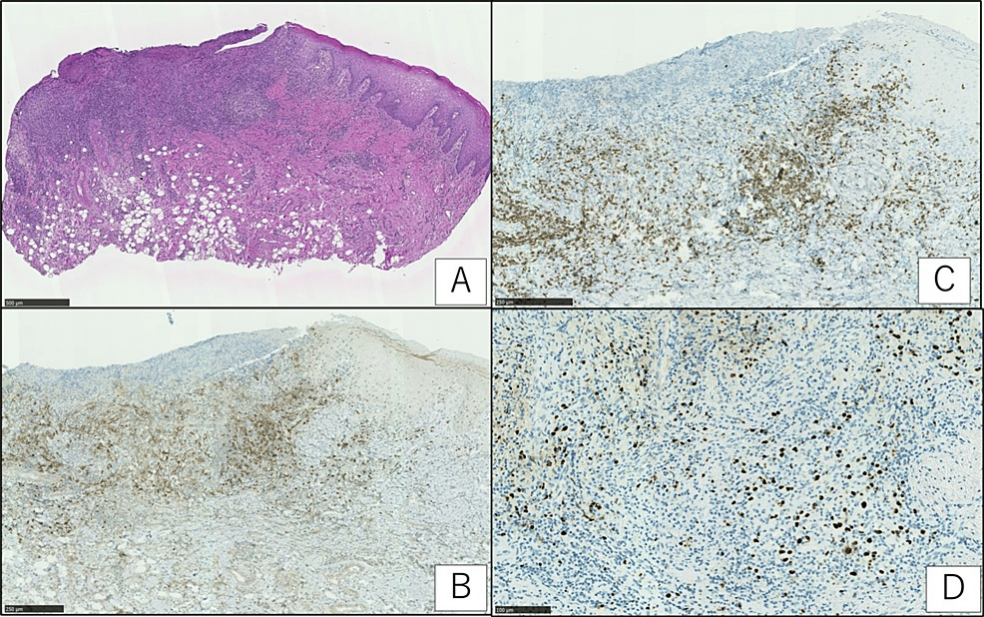
Histopathological examination image of the lesion (A): low magnification of an ulcer on the tongue reveals a highly cellular lymphocytic infiltrate deep into the submucosal layer (hematoxylin and eosin (H&E staining, scale bar = 500 µm); (B-C): the cells were B-cell in origin, being immunoreactive to CD20 (as seen in B; brown signals; bar = 250 µm) and positive for CD30 (as seen in C; brown signals; scale bar = 250 µm); (D): they were shown to be positive for Epstein-Barr virus-encoded early RNA via in situ hybridization (dark blue signals; EBER staining; scale bar = 100 µm). RNA: ribonucleic Acid; EBER: Epstein-Barr encoding region

Since the tongue lesion was refractory, malignancy was considered. The tongue lesion was performed by incisional biopsy. Atypical lymphocytes infiltrating the ulcer base were positive for CD20 and CD30. Atypical lymphocytes were positive for Epstein-Barr encoding region (EBER) in situ hybridization. Differential diseases included refractory stomatitis and malignant tumors. Based on the patient’s historical, clinical, and histopathological features, a diagnosis of EBVMCU was made (Figure 2A-2D).

**Figure 3:**
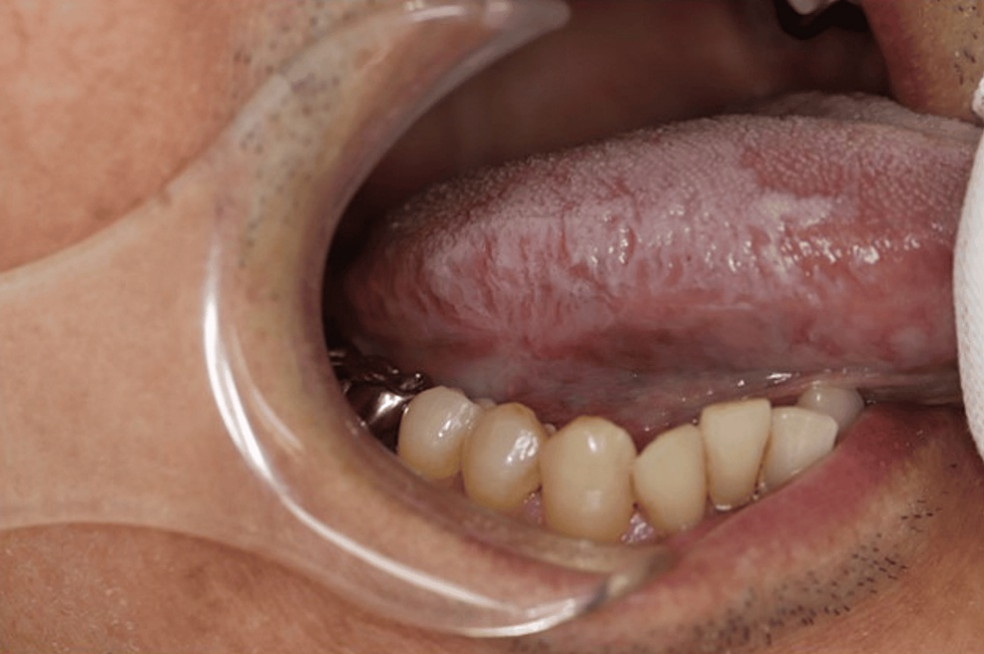
Image of an ulcer on the right lateral border of the tongue two months after the discontinuation of tacrolimus Ulcerative lesions healed. The white lesions on the lateral borders were due to candidiasis.

As interstitial pneumonia was well controlled, tacrolimus was discontinued, and the patient was switched to prednisolone monotherapy after obtaining sufficient patient consent. The tongue lesions healed two months after the discontinuation of tacrolimus (Figure 3).

## Discussion

Epstein-Barr virus is a DNA virus belonging to the family Herpesviridae that infects oropharyngeal epithelial cells and B-cells via the saliva, causing a latent infection of B-cells that can be lifelong [8,9]. More than 95% of adults are infected with EBV. Although most cases are subclinical, the use of immunosuppressive drugs and age-related immunodeficiencies can cause EBV-infected B cells to reactivate and proliferate, leading to EBV-positive lymphoproliferative disease [3,10].

According to a report of 25 cases of EBVMCU that occurred in the oral cavity, the breakdown of factors responsible for the occurrence of EBVMCU included 24 cases of medical etiopathogenesis and one case of age-related etiopathogenesis [11]. The immunosuppressive drugs administered for medically induced EBVMCU included methotrexate in all cases and multiple drugs in 13 cases. No cases of EBVMCU caused by the combination of prednisolone and tacrolimus have been reported in the literature thus far, making this case extremely rare [11].

Ulcerative lesions occur on the tonsils, pharynx, tongue, palate, buccal mucosa, skin, sinuses, gastrointestinal tract, lungs, and eyelids, but most commonly within the oral cavity [1,5,11]. The reason for this distribution is the plentiful distribution of EBV-infected B-cells in Waldeyer’s pharyngeal ring and the fact that the oral cavity is an environment in which microscopic trauma to mucosal surfaces is likely to occur as a result of food residues and dentures [1,11]. The immunohistological features of EBVMCU include the proliferation of atypical lymphocyte-like cells of various sizes, the infiltration of various other cell types, and the appearance of Hodgkin/Reed-Sternberg-like cells [12]. Differentiating EBV-positive diffuse large B-cell lymphoma (EBV+ DLBCL) from classical Hodgkin’s lymphoma is challenging [13]. Systemic evaluation with positron emission tomography-CT (PET-CT) is necessary to distinguish EBVMCU from other diseases, and the presence of multiple sites of involvement or mass lesions may suggest the possibility of a systemic lymphoproliferative abnormality [11].

In addition, sIL-2R levels have been reported to be useful as sensitive markers for distinguishing EBV+ DLBCL from EBVMCU [14]. In cases of EBVMCU, sIL-2R levels have been reported to be lower than those in EBV+ DLBCL, and other malignant lymphomas. The mean sIL-2R value is 4,584 in EBV+ DLBCL, 652 in EBVMCU, and 2,392 in other malignant lymphomas [14].

In this case, PET-CT showed no fluorodeoxyglucose accumulation throughout the patient’s body, and his serum sIL-2R level was 942 U/mL, similar to that of the EBVMCU case reported by Ikeda et al. [14]. Although a standard treatment for EBVMCU has not yet been established, the disease is believed to respond well to conservative treatment, such as a reduction or discontinuation of immunosuppressive drugs [1,5]. Antibody therapy targeting CD20 or CD30, local radiation, surgical resection, and chemotherapy are the treatments of choice for patients who cannot be weaned off of immunosuppressive drugs or are refractory [5,15]. With these treatments, remission occurs in 80% of all EBVMCU cases, and cause-related deaths are reported in only 1% [16].

In the present case, FK506 was discontinued and prednisolone was continued following consideration of the presence of interstitial pneumonia. In this case, reduction of the immunosuppressive drugs resulted in healing of the lesion. This suggests that, in cases of EBVMCU caused by multiple immunosuppressive drugs, switching to monotherapy instead of discontinuing all immunosuppressive drugs may be useful.

## Conclusions

EBVMCU generally has a benign course and may be cured by the reduction or discontinuation of any immunosuppressive drugs that may have caused the disease. In cases where patients take multiple immunosuppressive medications, EBVMCU can be cured while continuing the treatment of the primary disease by reducing the treatment to a single immunosuppressive medication.
